# Effect of Community Nutrition Rehabilitation Using a Multi-Ingredient Flour on the Weight Growth of Moderately Acute Malnourished Children in Benin

**DOI:** 10.3390/foods12020263

**Published:** 2023-01-06

**Authors:** Flora T. F. Lalèyè, Nadia Fanou-Fogny, Flora J. Chadare, Yann E. Madodé, Polycarpe A. P. Kayodé, Djidjoho J. Hounhouigan

**Affiliations:** 1 School of Nutrition and Food Sciences & Technologies, Faculty of Agronomic Sciences, University of Abomey-Calavi, Cotonou 01 BP 526, Benin; 2 Laboratory of Food and Bioresource Sciences and Technologies and Human Nutrition, School of Sciences and Techniques of Conservation and Transformation of Agricultural Products, University Center of Sakété, National University of Agriculture, Sakété 05 BP 1752, Benin

**Keywords:** malnutrition, food supplements, multi-ingredient porridge, LNR sessions, nutritional status

## Abstract

Childhood malnutrition remains a public health problem in Benin. This study aimed to assess the nutritional potential of complementary food resources to accelerate the weight growth of moderately malnourished children hosted in learning and nutritional rehabilitation centers (LNRs) in eight municipalities in Benin. A multi-ingredient infant flour (i.e., FARIFORTI), composed of 35% corn flour (*Zea mays*), 15% malted sorghum (*Sorghum bicolor*), 30% soybean (*Glycine max*), 10% shelled and roasted peanuts (*Arachis hypogeaea*), 7% baobab pulp (*Adansonia digitata*), and 2% dried fried fish (*Stolothrissa tanganyicae*), was tested with 289 moderately malnourished children aged 6 to 59 months, selected in LNR sessions. Children were given the FARIFORTI flour porridge over 12 days (based on LNR protocol) in addition to other dishes based on local food resources. The weight and height of the children were measured at entry and at the end of the LNR sessions. The sensory evaluation indicated that the FARIFORTI flour was well-accepted by mothers (97%) and children (98%). The FARIFORTI porridge provided significantly higher intakes of carbohydrates and iron in children with weight gain compared to children without weight gain.

## 1. Introduction

Childhood malnutrition, including the global, chronic, and acute types, as well as micronutrient deficiencies, remains a public health problem in Benin [1]. Childhood malnutrition was responsible for 96 deaths out of 1000 births of children aged 0 to 59 months, between 2001 and 2017, with 32% suffering from chronic malnutrition, 5% from global acute malnutrition, and 17% underweight and with worrying rates of iron, zinc, and vitamin A and C deficiencies [2]. In children under 5 years, moderate acute malnutrition is defined as a mid-upper arm circumference (MUAC) of 115 mm ≤ MUAC < 125 mm and a weight-for-height between −3 and −2 Z-scores of the median of the WHO child growth standards without edema [3].

From six months of age, breast milk is no longer sufficient for children’s nutritional quality and quantity. In order to cover their nutritional needs, mothers gradually introduce complementary foods into children’s rations to accompany breast milk [4]. In Benin, 28 and 15% of children aged 6 and 23 months, respectively, have achieved the minimum acceptable diet [3]. The most frequent foods given to children from 6 to 23 months are generally solid or semi-solid (58% in breastfed children and 89% in non-breastfed children) and liquid such as drink-based juices, light flavored broths, and other non-milk liquids (44% in breastfed children and 61% in non-breastfed children) [3]. The first complementary foods introduced are mainly porridge made from cereals, legumes, and/or tubers [5,6,7,8]. Protein intake from these porridges, whether simple or mixed with other ingredients, is often of low biological value and does not adequately cover the needs of children of complementary age [5,9,10].

An inventory of supplement flours for complementary feeding sold in pharmacies and drugstores showed that in urban areas of Benin, available flours do not comply with the standards of the Codex Alimentarius in terms of nutritional composition (i.e., protein content < 15 g/100 g; vitamin A < 140 μg/100 g; iron < 16 mg/100 g), and an energy content < 400 Kcal/100 g) [11]. In addition, the recommended macronutrient proportions by WHO are 12% dry basis protein with 6% animal protein and 6% vegetable protein. In the flours inventoried, the protein intake is mainly of plant origin [12]. Moreover, at the household level, mothers often take the flour from the family cereal flour stock (usually single maize flour), and cook it as porridge for the children without any additional nutritious foods [10]. The microbiological quality of these home preparations does not comply with the WHO recommendations [13]. This can be the cause of infectious diseases, which together with the poor nutritional quality, strongly contribute to the deterioration of the nutritional status of children at a complementary feeding age [14].

Taking into account these limits, a multi-ingredient flour (i.e., FARIFORTI) from locally available resources, meeting microbiological and health standards, was developed within the INFLOR project (Infant Foods from Local Resources as a pathway to a better food and nutrition security in Benin), using the Codex standard CXS 74-1981 for processed cereal-based foods for infants and young children [15]. The present research aimed to assess the effect of FARIFORTI flour on the weight growth of moderately acutely malnourished children in community care.

In Benin, the LNR (learning and nutrition rehabilitation center) approach is currently used for the management of moderate and severe acute malnutrition without complication (MAM and SAM) at the community level [16]. The LNR approach has as an aim, the nutritional recovery of MAM and SAM children after two weeks of treatment, using an optimal combination of locally accessible foods coupled with strategies to improve children’s appetites and nutrition education for effective behavioral change in nutrition, hygiene, and health [17]. This approach is based on the positive deviance approach. It involves mothers of children aged 6–59 months, whose particular practices and behaviors enable them to keep their children in good nutrition and health status, compared to their neighbors, while having access to the same resources and facing the same risk factors. They are called model mothers, often aged at least 16 years old, no older than 50, and in good health. Other criteria for selection involve their acceptance by the community. They must be committed, active, and have good attitudes and good morality. They must also be willing to devote 30% of their time to the activities and live in the village. This approach used recently in Niger yielded a success rate of 90% [18]. A similar success rate was also obtained with the same approach in Guinea [19]. In addition, a local complementary food, known as “Pappa di Parma”, prepared with local ingredients, was very effective against MAM in Sierra Leone [6]. In Benin, the LNR approach is implemented by the Multisectoral Food, Health and Nutrition Project (PMASN), one of the national community nutrition programs. Using the LNR facilities and therapy, this research aims to evaluate the contribution of FARIFORTI flour integrated into the menu of the LNR centers for the correction of MAM in children aged 6 to 59 months.

## 2. Materials and Methods

### 2.1. Study Framework and Design

The INFLOR project is a multi-partner action research project implemented in several work packages including the inventory and mapping of local food ingredients available in agroecological zones for the formulation of complementary foods; formulation of complementary foods according to the standards using available local food resources; assessment of consumer perception and acceptability of the developed flour; capacity building and skills of small and middle enterprises (SMEs) for the production of formulated flour; and certification of the quality of optimized and tested flour [15]. This study represents the experimental research phase of the INFLOR project. Ethical clearance was obtained from the National Ethics Committee for Health Research of Benin (CNERS): Ethical Clearance Code No. IRB00006860. Children’s parents were well informed, and we obtained their signed informed consent form before starting the study. The experiment was set up and monitored by a team of well-trained nutritionist assistants under the control of two supervisors and the general supervision of the principal investigator. 

The research design was an experimental crossover within-subjects design [20] with moderately malnourished children aged 6 to 59 months. We use the LNR facilities of the PMASN community nutrition program, as well as its therapy approach. Screening for malnutrition is organized by the PMASN program and children recruited as moderately malnourished undergo LNR therapy mainly based on LNR learning sessions, therapeutical feeding approach using local resources during 12 days, children nutritional status follow-up, etc. [17]. In our research design, each child represents his/her own control and, therefore, all children received fortified both FARIFORTI flour and the other local foods of the LNR therapy. The main endpoint was the weight gain of the children after 12 days of feeding therapy in the LNR centers [18].

This is a cross-over design study; however, as in many fields experiment surveys, the design was contextualized, making it difficult to have a full randomization. 

The first level of contextualization was the LNR settings, specifically reserved for acute malnourished children without complication. At this step, a full randomization was not possible, as we worked systematically with the LNRs running rehabilitation activities at the period of our fieldwork.

The second level of contextualization was the selection of children: as the LNR is supposed to be organized the closest as possible to the living place of the children and their family, a random assignment of the children to the LNR is not applicable. Indeed a screening is organized in the community and the children in the case of AM without complication are systematically enrolled in the LNR program. A fixed number of 15 children are hosted by LNR setting in a commune. Exceeding this number, another LNR should be opened.

The third level of contextualization was the foods consumed by the children: the LNR feeding model is standardized, so we had to adapt our design to it. Randomization was not applicable here as well, as all children should get both the improved formula and the home intake, so that our supplement does not replace the home foods intake but complements it. In that case, each child’s feeding scheme is the same every day: our improved formula as breakfast and a meal prepared with local resources as lunch. The dinner is prepared at home in the evening when the mothers go home.

Briefly, the crossover design model was identified as the closest to our experimentation model, and also for statistical analysis. As said, both treatment A (improved infant formula) and B (foods from local resources) are given on the same day. In our context, the watch out period is set at the 12th day of the LNR, when the children are expected to meet the required gain weight. However, we did not have the opportunity to go through the period 2 as the whole procedure for opening a new LNR session should be run again (screening at community level, identification of AMC without complication, preparation of LNR sessions, etc.).

### 2.2. Study Area

**Figure 1:**
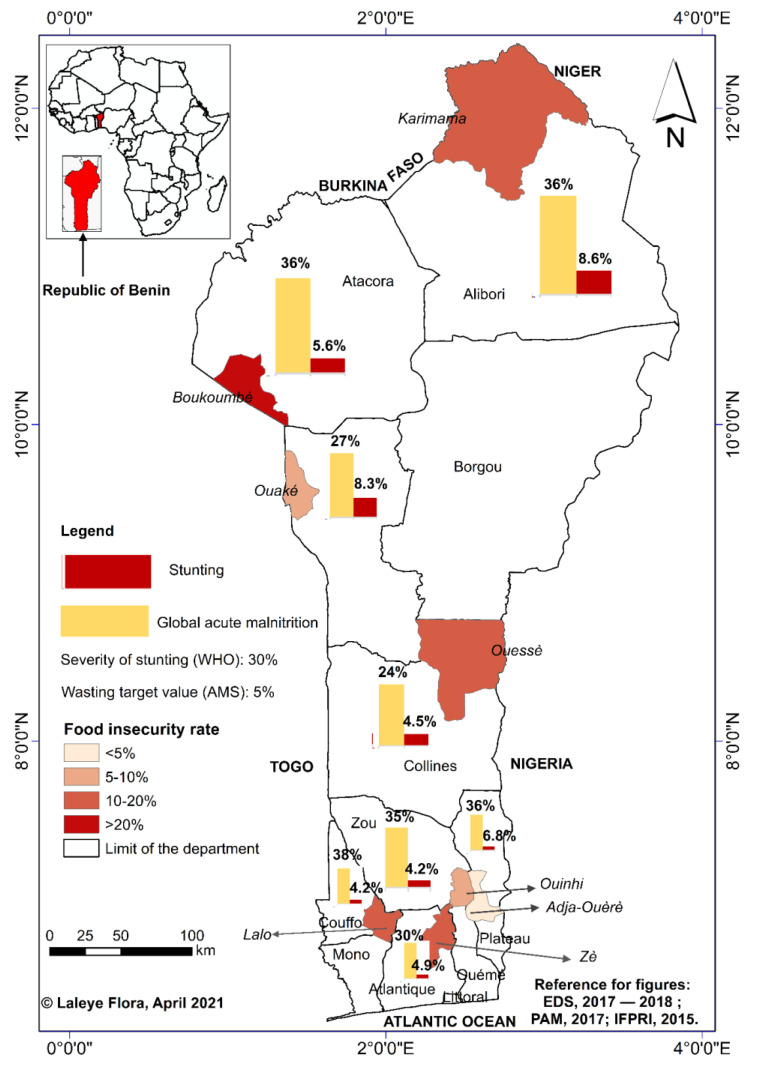
Study areas.

The study was conducted in Benin departments where the prevalence of stunting exceeded the threshold of 30%, according to the WHO [21], and where global acute malnutrition (AM) had reached or was close to the 5% target [22] (Figure 1). These were the departments of Alibori (stunting: 36%; AM: 8.6%), Atacora (stunting: 36%; AM: 5.7%), and Donga (stunting: 27%; AM: 8.3%) in northern Benin; Zou (stunting: 35%; AM: 4.2%) and Collines (stunting: 24%; AM: 4.5%) in the center of Benin; and Couffo (stunting: 38%; AM: 4.2%), Plateau ( stunting: 36%; AM: 6.8%), and Atlantic (stunting: 30%; AM: 4.9%) in southern Benin [2]. In each department, the intervention was implemented in the functional LNR centers at the time of the study; thus, in total, there were 22 LNR centers distributed among the municipalities of Karimama, Boukoumbé, Ouaké, Ouinhi, Ouèssè, and Adja-Ouèrè [21].

### 2.3. Participants and Sampling

Children aged 6 to 59 months who were moderately acute malnourished (MAM) were the main target of this study. A sample size of 289 was estimated as sufficient to detect a minimum weight difference of 200 g, considering a power of 99%, a significance level of 5%, and a nonresponse rate of 5% [23]. MAM children were identified by community screening for malnutrition organized by the PMASN project. We worked with children aged 6 to 59 months old and suffering from uncomplicated MAM, and admitted to LNR centers during the study period. Exclusion criteria for our study was: suffering from diseases, such as diabetes, hypertension, and coeliac disease (allergy to wheat flour), and allergy to any other component of the multi-ingredient flour FARIFORTI. There were no excluded children in our sample. According to the LNR protocol, a learning session must host a maximum of 15 malnourished children [24]. Table 1 shows the distribution of the children among the 22 LNR sessions.

### 2.4. LNR Learning Sessions

Following the LNR approach guide, each learning session lasted 12 days and was led by “model mothers”, who were women identified by the community [24]. Each daily learning session started in the morning at 10 o’clock for a duration of 3.5 h to 4 h as follows: a complementary food cooking session using local food resources (1 h), early child development games and children’s feeding therapy (1 h to 1.5 h), an awareness communication session after site cleaning (1 to 1.5 h). For the experimentation, the field assistants worked closely with the model mothers during the sessions. 

### 2.5. Experimental Foods

For the experimentation, two categories of complementary foods were considered: the FARIFORTI flour porridge (i.e., experiment food) and the other foods prepared during the LNR sessions. The FARIFORTI flour and the porridge recipe were developed and standardized as part of the INFLOR project [15]. The ingredients were identified from a bibliographical review and an ethnobotanical study on the local food resources available by agroecological zone and potentially usable in communities for the preparation of children’s complementary foods [25].

### 2.6. FARIFORTI Flour Porridge

The FARIFORTI flour was composed of 35% corn flour (*Zea mays*), 15% malted sorghum (*Sorghum bicolor*), 30% soybean (*Glycine max*), 10% shelled and roasted peanuts (*Arachis hypogeaea*), 7% baobab pulp (*Adansonia digitata*), and 2% dried fried fish (*Stolothrissa tanganyicae*) (100 g on a dry matter basis). During the LNR sessions, the porridge was prepared in bulk each day by the assistants using a quantity of 750 g of flour dissolved in 5.30 L of water. Every day during the session, each child received a portion size of 50 g of the prepared porridge. The assistants made sure that each child consumed the entire served portion without any leftovers.

### 2.7. Other Complementary Foods

Three daily menus made of local food resources were prepared alternately over the 12 days of the LNR sessions: white or red cowpeas with peanut oil or red palm oil and fried fish; mixed cowpeas with rice (*atassi* in *fongbe*, the local language) accompanied by peanut sauce or fried fish; mixed cowpeas with corn flour and red palm oil and fried fish (*djongoli* in *fongbe*, the local language); mashed sweet potato/cassava accompanied by fried fish; boiled Bambara nuts with red palm oil; cooked rice with peanut sauce; red (with tomato) fatty rice with egg; corn or millet or rice flour dough, accompanied by different sauces made of either peanut, palm nut, okra, baobab, moringa, tomato, *corchorus* leaves (jute mallow) or cassava leaves. Every day, in addition to the FARIFORTI porridge, children consumed one of these dishes. Oranges, citrus, and other available fruits were served to children either as a drink or were directly consumed.

### 2.8. Sensory Test

A hedonic test was carried out with different children and mothers to determine the sensory acceptability of the FARIFORTI porridge. The porridge was submitted to the assessment of the panel of mothers and children. A five-point Likert scale, ranging from 1 (“don’t like at all”) to 5 (“like a lot”), and 3, where indifference was used to assess the porridge for consistency, color, and taste. The children’s facial expressions were also noted after tasting [26]. The sensory test of the improved porridge was carried out on the first day of consumption. The assessment was made using a descriptive test. The mothers gave their perception of the taste, color, and consistency of the porridge and then their overall appreciation on a scale of five levels from “dislike a lot” to “like a lot” [26,27]. As for the children, their facial expressions were assessed when eating the porridge, also on a scale of five levels, from “dislike a lot” to “like a lot” [26,27]. Acceptability by the children was also measured daily by the amount of porridge remaining at each FARN session. As the porridge was an improved formula from that usually consumed by children, total rejection by children was not expected at the end of this phase. Sugar was also added when consuming the porridge.

### 2.9. Anthropometric Measurement and Food Consumption Data

Weight: The children’s weight was measured according to standard procedures using a UNICEF electronic scale (capacity: 25 kg; accuracy: 100 g) [28]. The scale was calibrated every morning using a 5 kg standard weight before measurements. Each child was weighed twice, completely undressed.

Height: The height of the children was determined according to standard procedures [28], using UNICEF’s height and length measuring board made of wood graduated in centimeters. It was placed either horizontally or vertically to measure the child standing (older than two years) or laying down (younger than two years). Each child was measured twice. 

Mid-upper arm circumference (MUAC): This was measured according to standard procedures [28] with an MUAC tape. The tricolored bracelet had a precision of 0.1 cm.

Food consumption data: Children’s food intake was measured at baseline and at the end of the LNR learning sessions using the weighed food record method. At each session, all food consumed by the children (including the FARIFORTI porridge) was weighed by the assistants before the children consumed the food, with the help of the mothers. To determine the intakes, each ingredient used to cook the food was weighed before being cooked, and the whole cooked meal was weighed in bulk. The portions consumed of each ingredient was calculated from the ratio of the amount consumed by the children to the total amount cooked. 

### 2.10. Data Analysis

Anthropometric indices (Z-score) were calculated with ENA for SMART software version 2011 (SMART Technologies ULC, USA), using the measured weight and height (W/H). These were weight-for-height, height-for-age (H/A), and weight-for-age (W/A) Z-scores. The standard minimum weight gain for a successful rehabilitation was 200 g after 12 days of treatment in LNR sessions, with an average acceptable minimum weight gain of 3 g/kg/day for an average of 10 to 20 g/day [19]. Daily nutrient intake (i.e., energy, protein, iron, and vitamins A and C) was determined using the food consumption data and the West African food composition table [29]. A simple descriptive analysis was used to compute the mean and standard error (SE) and to draw the graphs for the changes in weight gain. Student’s *t*- or chi-square tests were performed for the mean or proportion comparison by sex and age class for the sample’s characteristic variables. The paired Wilcoxon test was computed, due to non-normality, to compare the means of the z-scores before and after the LNR sessions. Logistic regression was used to estimate the respective contribution of the FARIFORTI porridge and other foods to weight gain. The dependent variable is: the weight gain and the independent variables are intakes respectively from FARIFORTI flour and other foods. Data were analyzed with SPSS software (SPSS Version, 21.0. Armonk, NY, IBM Corp). Values with *p*-values < 0.05 were significant.

## 3. Results

### 3.1. Children’s Characteristics

The share of children by sex was similar, with 58% being girls, and a 0.7 sex ratio of girls/boys (Table 2). The mean age of the children was 24.63 months (95% CI: 23.05, 26.20). More than half of the children (57.4%) were aged between 6 and 23 months old, and the mean age did not vary with sex. The respective mean weight, height, and MUAC of the children were 8.78 kg (95% CI: 8.53, 9.03), 78.22 cm (95% CI: 77.16, 79.28), and 29 cm (24.53, 33.69), and they did not vary significantly by sex. By contrast, the Z-scores for the W/H, H/A and W/A for the girls were −1.53 (95% CI: −1.78, −1.28), −2.11 (95% CI: −2.38, −1.84), and −2.29 (95% CI: −2.50, −2.09), which were significantly lower than for the boys, at −1.71 (95% CI: −1.93, −1.50), −2.61 (95% CI: −2.94, −2.28), and −2.61 (95% CI: −2.80, −2.41), respectively.

### 3.2. Acceptability of the FARIFORTI Flour

Overall, all mothers appreciated the FARIFORTI porridge: 97% liked it a lot, 93% for the consistency, 96% for the color, and 81% for the taste. None of mothers disliked or remained neutral to the porridge, and no child showed any sign of allergy or refusal.

### 3.3. Anthropometric Indices before and after the LNR Sessions

**Figure 2:**
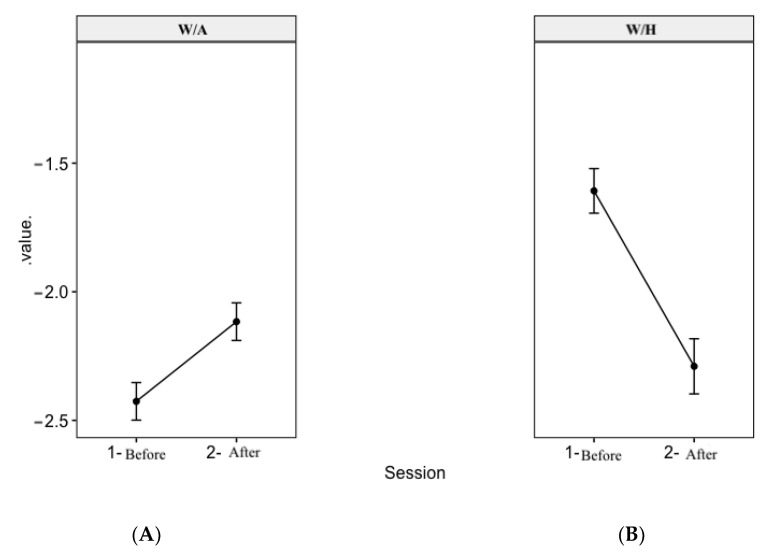
Changes in the (**A**) weight-for-age (W/A) and (**B**) weight-for-height (W/H) Z-scores of the MAM children before (day 1) and after (day 13) the LNR experimental sessions (*n* = 289). Wilcoxon test.

The respective mean weight-for-age (W/A) and height-for-age (H/A) Z-scores at the end of the LNR sessions were −2.12 (95% CI: −2.26, −1.97) and −1.18 (95% CI: −1.36, −1.01), similar to +1 SD of the baseline Z-scores of −2.43 (95% CI: −2.57, −2.28) and −2.32 (95% CI: −2.53, −2.11) (Figure 2). This indicates a decrease in the prevalence of underweight and stunting at the end of the LNR sessions. On the contrary, the mean weight-for-height (W/H) Z-score at the end of the LNR sessions (−2.29 (95% CI: −2.50, −2.08)) was more than +1 SD of the baseline value (−1.61 (95% CI: −1.78, −1.44)). This indicates that the prevalence of acute malnutrition did not decrease after the LNR sessions. In addition, we can notice the difference of W/A and W/H before and after the LNR sessions are both significant (*p* < 0.001).

### 3.4. Weight Growth of Children after the LNR Experimental Sessions

**Figure 3:**
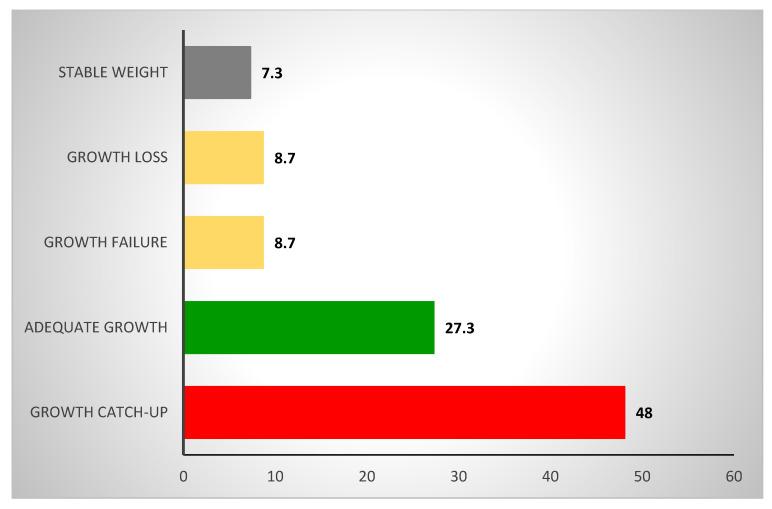
Weight growth of the children at the end of the LNR sessions (*n* = 289). Appropriate weight gain: ≥400 g; stable weight: weight gain equal to their weight at admission; adequate growth: weight gain ≥200 and ≤400 g; weight loss: weight gain ˂ admission weight; growth failure: ˂200 g).

The children’s weight gain ranged from 40 to 2300 g over the two weeks of the LNR sessions, with an average of 425.35 (±18.91) g corresponding to an average daily gain per child of 35.45 g/day. This weight gain was greater than 10 to 20 g/day per child, which is considered necessary for successful nutritional rehabilitation. The weight growth of children varied significantly from the start to the end of the LNR sessions (*p* < 0.001). At the end of the LNR sessions, 21 (7.30%) had a stable weight (equal to their weight at admission), 25 (8.7%) did not gain enough weight (weight gain <200 g), 79 (27.3%) recovered an adequate growth (weight gain ≥200 and ≤400 g) and 139 (48%) of the children were completely rehabilitated (weight gain ≥400), which indicated a success rate of 75.4% (Figure 3).

### 3.5. Energy and Nutrient Intake from Foods Consumed during the LNR Sessions

There was a significant difference between energy and nutrient intakes from foods with and without the FARIFORTI porridge during the LNR sessions (Table 3). In regard to energy and macronutrients, the FARIFORTI porridge contributed significantly to 45% of the energy intake and 30% of the protein intake of the LNR meals. The contribution of micronutrients by the FARIFORTI porridge was much less: 11% for iron, 8% for vitamin C, and less than 1% for vitamin A.

There was no significant difference between children who recovered weight and those who did not for energy intake from the FARIFORTI porridge and other foods consumed by the children (Table 4). The FARIFORTI porridge provided significantly higher intakes of carbohydrates and iron in children with weight gain compared to children without weight gain (*p* < 0.05), while the other foods (without the FARIFORTI porridge) provided significantly more vitamin C only in children with weight gain compared to children without weight gain (*p* < 0.05).

## 4. Discussion

Improving infant nutrition requires food systems that provide nutritious, safe, affordable, and sustainable food for all children [3]. The present study aimed to assess the effect of complementary foods based on local resources on the weight growth of moderately acute malnourished (MAM) children aged 6 to 59 months who attended LNR sessions.

The multi-ingredient porridge (FARIFORTI) based on local food resources was preferred and accepted by mothers and children, and it is likely to contribute effectively to weight gain. This result is similar to that obtained by Borg et al. [30] in Cambodia but contrary to that of Azimi et al. [31], who found that the formulation of supplementary foods made it possible to reduce the prevalence of malnutrition acute moderate (MAM) in Iran. The non-decrease in the prevalence of MAM in this case would be related to the short period of the LNR sessions. Azimi et al. [31] took eight weeks to improve the nutritional status of MAM children with a food supplement based on local food resources in Iran.

The weight gain in the children in this study was not consistently related to energy intake from foods consumed by the children. All children who gained weight tended to have more total carbohydrates, iron, and vitamins. Weight gain ranged from 40 to 2300 g for the two weeks with an average of 425.35 (±18.91) g per day, which corresponded to a daily gain for each child of 35.45 g/day. This weight gain was greater than 10 to 20 g/day per child, which is considered necessary for good nutritional rehabilitation [19]. This result reflected the nutritional quality of the porridge, other foods, and the adequacy of the foods for the children’s eating habit. However, weight loss varied from 100 to 900 g over the two weeks, and an average of 276.00 (±44.45) g per day was observed. Moreover, a stable weight, lost weight, adequate growth experienced, and a catch-up situation were also observed. These results would be due to the fact that the children examined were beginning to become familiarized to foods other than breast milk and/or did not like acidic foods very much (i.e., the FARIFORTI porridge) [32]. As children were in a complementary feeding age, mothers do continue breastfeeding their toddlers but we did not quantify this in our study.

Compared to those who did not gain adequate weight, the metabolism of total carbohydrates from the FARIFORTI porridge in the bodies of the children greatly contributed to their weight gain. This rapid assimilation by the children bodies would be due to the fact of biotechnology processes, such as malting and fermentation, which were used to develop the flour, and the various raw materials and ingredients used to produce this multi-ingredient flour. Flour is, in fact, mainly composed of cereals (i.e., corn and sorghum), legumes (i.e., soybeans and peanuts), dry fruit pulp (i.e., baobab), and fried fish. The macronutrient content of this complementary flour, compared with the recommended values of the Codex Alimentarius, provided 100% (498.72 Kcal/100 g) of the energy and more than 100% of proteins (15 and 10 g/100 g).

The lipid content was within the range of the recommended values (13 and 42 g/100 g), the fiber content was <5 g/100 g, as recommended, and the theoretical iron content of the flour was 3.12 mg/100 g [10]. The advantage of incorporating soy into the weaning porridge was justified by the fact that soy contains a balanced proportion of proteins of good biological value containing all essential amino acids as well as vitamins and minerals [7]. Its high lipid content gives it a high calorific value. The presence of baobab pulp is an important source of micronutrients. The process of processing raw materials and ingredients contributes to the acceptability of supplementary foods [4]. In fact, the various intermediate products involved in the constitution of the flour such as sorghum malt, hulled sorghum, hulled roasted soybeans and sifted baobab pulp were mixed to obtain a flour. This flour after humidification and stirring underwent a fermentation from 0 to 72 h. It was then dried and ground, to obtain the multi-ingredient flour. 

From the various results of this study, it should be noted that the daily consumption of this improved porridge associated with a recovery program allowed for a better improvement in the nutritional status of children with moderate emaciation, growth retardation, and underweight status. Regarding other LNR dishes consumed by children, the food groups that constituted them were also cereals (i.e., maize, rice, cassava, millet, and sorghum), legumes and nuts (i.e., soy, peanuts, and cowpea), meat, and fish. The other LNR dishes consumed by children also reinforce the daily consumption of multi-ingredient porridge. The nutritional benefits of consuming these food groups, in general, by children has already been noted in many studies. They provide children with minerals, vitamins, and nutritional factors that are often lacking in their diet.

The sampling of moderately malnourished children for this study included more female than male children with different age groups. This choice is similar to the work of Millimono et al. [19] on the assessment of the care of moderately acute malnourished children from 6 to 59 months in the learning and nutritional rehabilitation centers of Kouroussa prefecture. Millimono et al. [19] reported 54% girls and 46% boys in their study with a sample size of 322 children. Effectively, sex and age are biological parameters of differentiation of child status, which influence their diet and health according to the social value and beliefs [33]. According to these same authors, the rate of malnutrition increased from birth to 24 months and reversed rapidly after 24 months due to underweight and wasting.

However, it should be noted that this study has limitations. In reality, what must be remembered is that this treatment protocol chosen for MAM in FARN does not clearly allow the resolution of MAM but rather a weight gain. This is what our study shows. Additionally, note that the exit criterion for MAM children is essentially based on weight gain, which must be greater than or equal to 400 g.

## 5. Limits

For the robustness of the statistical analysis, this study should have included a washout period before an intervention period to evaluate the effect of the FARIFORTI porridge consumption over two periods on the weight growth of moderately malnourished children.

## 6. Conclusions

The study on the effect of complementary foods based on local food resources introduced at the LNR level has shown their beneficial effect on the weight gain of moderately malnourished children and their effective contribution in reducing the prevalence of underweight in children younger than five years old. The FARIFORTI multi-ingredient flour, due to its organoleptic characteristics, was highly appreciated by the children and their mothers. It brought more total carbohydrates, iron, and vitamin C into the body to induce weight gain in moderately malnourished children. Thus, these nutritional rehabilitation initiatives, which largely consider local foods, must be perpetuated. However, a very rigorous follow-up in the implementation of the recipes would be an asset for a positive reversal of the nutritional situation. Finally, the strategies of interventions using the FARIFORTI multi-ingredient flour in the LNR sessions should be promoted to combat children’s acute malnutrition.

## Figures and Tables

**Table 1:** Number of infants in the study (*n* = 289).

Regions	Commune		
		*n*	%
South	Adja-Ouèrè	31	10.7
	Lalo	32	11.1
	Zè	30	10.4
	Total Southern Benin	93	32.2
Center	Ouèssè	24	8.3
	Ouinhi	30	10.4
	Total Central Benin	54	18.7
North	Boukoumbé	41	14.2
	Karimama	53	18.3
	Ouaké	48	16.6
	Total Northern Benin	142	49.1
Total		289	100

*n* = number of infants who participated in the study. % = infant participation rate.

**Table 2:** Baseline characteristics of the MAM children participating in the LRN learning sessions.

Characteristics	Total (*n* = 289)	Girls (*n* = 167)	Boys (*n* = 122)	*p*-Value *
Sex (%)		57.8	42.2	0.974 ^a^
age (months)				0.774 ^b^
Average (SE)	24.63 (0.80)	25.11 (1.11)	23.97 (1.14)	
95% CI	(23.05, 26.20)	(22.92, 27.30)	(21.71, 26.22)	
age class (%)				0.944 ^a^
(6–24)	166 (57.4%)	95 (56.9%)	71 (58.2%)	
(24–36)	61 (21.1%)	35 (21.0%)	26 (21.3%)	
(36–60)	62 (21.5%)	37 (22.2%)	25 (20.5%)	
weight (kg)				0.379 ^b^
Mean (SE)	8.78 (0.13)	8.70 (0.18)	8.89 (0.18)	
95% CI	(8.53, 9.03)	(8.35, 9.06)	(8.53, 9.24)	
height (cm)				0.958 ^b^
Mean (SE)	78.22 (0.54)	78.35 (0.75)	78.04 (0.77)	
95% CI	(77.16, 79.28)	(76.87, 79.83)	(76.52, 79.56)	
MUAC (cm)				0.893 ^b^
Average (SE)	29.11 (2.33)	29.70 (3.13)	28.31 (3.49)	
95% CI	(24.53, 33.69)	(23.52, 35.88)	(21.41, 35.21)	
weight-for-height				0.031 ^b^
Average (SE)	−1.61 (0.09)	−1.53 (0.13)	−1.71 (0.11)	
95% CI	(−1.78, −1.44)	(−1.78, −1.28)	(−1.93, −1.50)	
height-for-age				0.009 ^b^
Average (SE)	−2.32 (0.11)	−2.11 (0.14)	−2.61 (0.17)	
95% CI	(−2.53, −2.11)	(−2.38, -1.84)	(−2.94, −2.28)	
weight-for-age				0.001 ^b^
Average (SE)	−2.43 (0.07)	−2.29 (0.10)	−2.61 (0.10)	
95% CI	(−2.57, −2.28)	(−2.50, −2.09)	(−2.80, −2.41)	

SE: Standard error. ^a^ χ^2^ test; ^b^ Student’s *t*-test. * between boys and girls.

**Table 3:** Energy and nutrient intakes during the foods consumed by the children in LNR centers with and without the FARIFORTI porridge (*n* = 289).

	Foods Without FARIFORTI	Foods With FARIFORTI	*p*-Value
Energy (kJ)			<0.001
Mean (SE)	11,004.33 (414.20)	20,105.26 (599.28)	
95% CI	(10,189.13–11,819.53)	(18,925.74–21,284.78)	
Protein (g)			<0.001
Mean (SE)	134.80 (48.44)	193.15 (49.10)	
95% CI	(39.47–230.14)	(96.50–289.80)	
Fat (g)			<0.001
Mean (SE)	134.82 (45.77)	175.14 (46.43)	
95% CI	(44.73–224.91)	(83.77–266.52)	
Carbohydrates (g)			<0.001
Mean (SE)	375.05 (47.46)	507.31 (48.29)	
95% CI	(281.65–468.45)	(412.25–602.36)	
Iron (mg)			<0.001
Mean (SE)	197.61 (56.67)	225.24 (57.33)	
95% CI	(86.07–309.14)	(112.40–338.08)	
Vitamin A (µg)			0.958
Mean (SE)	1378.90 (91.12)	1378.93 (92.38)	
95% CI	(1199.60–1558.26)	(1197.07–1560.74)	
Vitamin C (mg)			0.409
Mean (SE)	125.93 (12.16)	135.63 (13.45)	
95% CI	(102.00–149.87)	(109.16–162.09)	

SE: Standard error. CI: confidence intervals.

**Table 4:** Energy and nutrient intakes from the FARIFORTI porridge and other foods consumed during the LNR learning sessions according to weight gain.

	No. of Children without Weight Gain (*n* = 46)	No. of Children Weight Gain (*n* = 243)	*p*-Value
FARIFORTI Porridge			
Energy (kJ)			0.823
Mean (SE)	10,231.46 (1470.56)	8816.43 (442.02)	
95% CI	(7269.59, 13,193.32)	(7945.73, 9687.12)	
Carbohydrates (g)			0.017
Mean (SE)	124.03 (5.46)	131.56 (2.05)	
95% CI	(113.04, 135.03)	(127.53, 135.60)	
Iron (mg)			0.042
Mean (SE)	19.09 (2.99)	26.25 (1.37)	
95% CI	(13.07, 25.11)	(23.55, 28.95)	
Other foods			
Energy (kJ)			0.855
Mean (SE)	10,940.88 (944.38)	11,086.83 (465.48)	
95% CI	(9038.81, 12,842.96)	(10,169.93, 12,003.74)	
Vitamin C (mg)			0.030
Mean (SE)	71.96 (16.66)	136.82 (14.23)	
95% CI	(38.41, 105.51)	(108.79, 164.85)	

## Data Availability

The data presented in this study are available on request from the corresponding author. The data are not publicly available due to the presence of personal information of the respondents.
